# Isolation and Analysis of the *Cppsy* Gene and Promoter from *Chlorella protothecoides* CS-41

**DOI:** 10.3390/md13116620

**Published:** 2015-10-28

**Authors:** Meiya Li, Yan Cui, Zhibing Gan, Chunlei Shi, Xianming Shi

**Affiliations:** 1 MOST-USDA Joint Research Center for Food Safety, School of Agriculture and Biology, and State Key Lab of Microbial Metabolism, Shanghai Jiao Tong University, Shanghai 200240, China; E-Mails: lmeiya@126.com (M.L.); cyan9028@163.com (Y.C.); forrestgzb@yahoo.cn (Z.G.); xmshi@sjtu.edu.cn (X.S.); 2 Analytical Testing Center, Zhejiang Chinese Medical University, Hangzhou 310053, China

**Keywords:** *Chlorella protothecoides* CS-41, phytoene synthase, *Cppsy*, promoter

## Abstract

Phytoene synthase (PSY) catalyzes the condensation of two molecules of geranylgeranyl pyrophosphate to form phytoene, the first colorless carotene in the carotenoid biosynthesis pathway. So it is regarded as the crucial enzyme for carotenoid production, and has unsurprisingly been involved in genetic engineering studies of carotenoid production. In this study, the *psy* gene from *Chlorella protothecoides* CS-41, designated *Cppsy*, was cloned using rapid amplification of cDNA ends. The full-length DNA was 2488 bp, and the corresponding cDNA was 1143 bp, which encoded 380 amino acids. Computational analysis suggested that this protein belongs to the Isoprenoid_Biosyn_C1 superfamily. It contained the consensus sequence, including three predicted substrate-Mg^2+^ binding sites. The *Cppsy* gene promoter was also cloned and characterized. Analysis revealed several candidate motifs for the promoter, which exhibited light- and methyl jasmonate (MeJA)-responsive characteristics, as well as some typical domains universally discovered in promoter sequences, such as the TATA-box and CAAT-box. Light- and MeJA treatment showed that the *Cppsy* expression level was significantly enhanced by light and MeJA. These results provide a basis for genetically modifying the carotenoid biosynthesis pathway in *C. protothecoides*.

## 1. Introduction

Lutein is one of more than 750 known naturally occurring carotenoids, and is synthesized by higher plants, bacteria, fungi, and algae. Based on its molecular structure (containing oxygen), it belongs to the xanthophyll family, one of the two major carotenoid families. In the plant kingdom, lutein provides photoprotection by scavenging singlet oxygen and peroxyl radicals [1], and its bright yellow color helps plants achieve effective cross pollination. Humans cannot synthesize lutein themselves, yet it is essential for the human body. Lutein is the predominant carotenoid in the infant brain [2], and is supplemented in newborn babies in the first hours of life. Lutein can increase biological antioxidant potential and reduce the plasma concentration of total hydroperoxides. It also reduces free radical-induced damage [3]. Lutein is the main carotenoid in the human retina; hence, it has been used as a therapeutic agent for the prevention of age-related macular degeneration [4,5]. Epidemiologic data suggest that lutein plays an active role in delaying chronic diseases [6], stimulating the immune response [7], and hampering the development of cataracts and atherosclerosis [8,9]. A recent study showed that a lutein-based dye used during chromovitrectomy in humans could improve the identification and removal of the vitreous, internal limiting membrane and the epiretinal membrane [10].

As lutein has many functions, it has become increasingly important to find and create more sources of lutein production. In recent years, algae have received a great deal of attention in the production of carotenoids and proteins. Previous studies in our laboratory showed that heterotrophically cultivated *Chlorella protothecoides* CS-41 can produce considerable amounts of lutein [11]. Furthermore, optimization of the cultivation conditions, medium composition, and extraction techniques can improve lutein yields [12,13]. However, to date, there are no reports of the enhancement of lutein production by this alga using genetic modification, although genetic engineering technologies have become increasingly popular in the field of carotenoid production. The first step is to determine the genes involved in lutein biosynthesis—information that is essential for genetic modification.

It has been found that phytoene synthase (PSY) is the rate-limiting enzyme in the carotenoid biosynthesis pathway in photosynthetic organisms [14,15,16]. In many cases, the rate of lutein formation through the carotenoid biosynthetic pathway appears to be controlled by PSY, which catalyzes the head-to-head condensation of two geranylgeranyl diphosphate molecules to yield phytoene—the first committed reaction in carotenogenesis. Since PSY plays a key role in the first step of carotenogenesis, it has unsurprisingly been chosen for genetic engineering studies of carotenoid production.

PSY has been extensively studied in bacteria and higher plants, but its study in algae is still in its infancy. For unicellular green algae, the *psy* gene has previously been investigated in *Chlamydomonas reinhardtii* [17], *Duanliella bardawil* [18], and *Haematococcus pluvialis* [19]. In *C. reinhardtii*, deletion of the *psy* gene resulted in a white phenotype [20]. For *Haematococcus*, *psy* was shown to be up-regulated under stress conditions of high light and low nutrient availability [21]. Overexpression of exogenous *psy* from *D. salina* [22] or *C. zofingiensis* [23] in *C. reinhardtii* has been shown to increase the lutein content to over 1.25 and 2.2-fold, respectively.

As an efficient lutein-production alga, *C. protothecoides* CS-41 has high potential for application in the commercial production of lutein; however, its lutein biosynthesis pathway has not been well studied. Our research group previously cloned other key enzyme genes in the lutein biosynthesis pathway of this alga, such as the phytoene desaturase (*pds*) (GenBank accession No. FJ968162) [24], zeta-carotene desaturase (*zds*) (GenBank accession No. GU269622) [25], and lycopene-ε-cyclase (*lyce*) (GenBank accession No. FJ752528) genes. The *psy* gene is essential for determining the complete lutein biosynthesis pathway in this alga. Therefore, in this study, the *psy* gene from the unicellular microalga *C. protothecoides* CS-41 and its promoter were isolated and analyzed. This study provides an important theoretical basis for the genetic modification of lutein biosynthesis in *C. protothecoides* CS-41, including gene sequences, expression promoter candidates, and possible regulatory environmental factors for gene expression.

## 2. Results and Discussion

### 2.1. Cloning and Characterization of the psy Gene from C. protothecoides

Touchdown PCR with primers YF and YR (Table 1) generated a predicted 373 bp fragment (Supplementary Figure S1, lane 1). BLAST analysis showed that the nucleotide sequence of this fragment shared about 74% and 73% identities with that of *C. reinhardtii* and *D. salina*, respectively, demonstrating that this fragment sequence is derived from a putative phytoene synthase.

**Table 1:** PCR primers and target fragments for *Cppsy.* F: forward; R: reverse; O: outer primer; I: inner primer.

Aim	Primer Sequence 5′-3′
**Partial *psy* fragment**	
YF	GCCATCTACGTGTGGTGCC
YR	CACGCAAGATGTTGGTCAGC
**5′-RACE-PCR**	
YFO1	GACTTGTCCACGCCCATCAC
YRI1	GGGGAAGCGGGAGATGGTGT
**3′-RACE-PCR**	
YFO2	GATGCTGCCCTCACAGACAC
YRI2	TGGATTTGGTCAAGTCACGC
**cDNA and DNA**	
YF1	ATGAGCACGTTTCTGAGCACAGTG
YR1	TCACATGCGCGCCCTCAG
**Probe**	
psy-F	GAAGTGACCAGCGAGTATGCC
psy-R	CTAAAGGGTTGGATGTGC
psyRTF	GAAGTGACCAGCGAGTATGCC
psyRTR	TCTCTAAAGGGTTGGATGTGC
**Promoter**	
PSYSP1	CTGTGCATGCGAAGTCGGAGTGAGA
PSYSP2	CGTCTTGGCATACTCGCTGGTCACTT
PSYSP3	ACTCATGCTGGGGGCTAGGAAAG
PSYSP1′	ATGGCGGGTGGCAGAGTCAATGTA C
PSYSP2′	CCAGACACAATCACCTCGCAGCCCTT
PSYSP3′	CGTTCACTCACCGCTCTCCATCACAA

With the sequence information, specific primers were designed for 5′- and 3′-rapid amplification of cDNA ends (RACE) of the related gene. 5′-RACE generated a 598 bp fragment (Supplementary Figure S1, lane 2), and 3′-RACE produced an 816 bp fragment (Supplementary Figure S1, lane 3). They were displayed by sequencing as the 5′ and 3′ regions of the phytoene synthase gene of *C. protothecoides* (*Cppsy*). RT-PCR (Reverse Transcription) with a pair of primers YF1 and YR1 generated an 1143 bp fragment (Supplementary Figure S1, lane 4), which was identified as the full-length *Cppsy* cDNA (GenBank accession No. FJ968161).

The open reading frame of *Cppsy* cDNA encoded a protein of 380 amino acid residues with a calculated molecular mass of 43.035 kDa and an isoelectric point of 6.40 (http://cn.expasy.org/tools/protparam.html) and shared 81.7% identical sequence with *Chlorella* NC_64A.

To characterize the corresponding gene of *Cppsy* cDNA, genomic PCR was performed. A 2488 bp fragment (Supplementary Figure S1, lane5) (GenBank accession No. GU351883) was generated and sequenced. Analysis of the obtained nucleotide sequence revealed that the product was the corresponding *Cppsy* gene.

The Southern blot analysis results indicated that there is only one *Cppsy* gene copy in *C. protothecoides* CS-41 (Supplementary Figure S2), which is different to those in higher plants. *Psy* gene replication is common in dicot plants, such as tomato (SlPSY1 and SlPSY2), and in monocot plants, such as maize (ZmPSY1-3), rice (OsPSY1-3), and sorghum (SbPSY1-3) [16,26,27,28].

**Figure 1:**
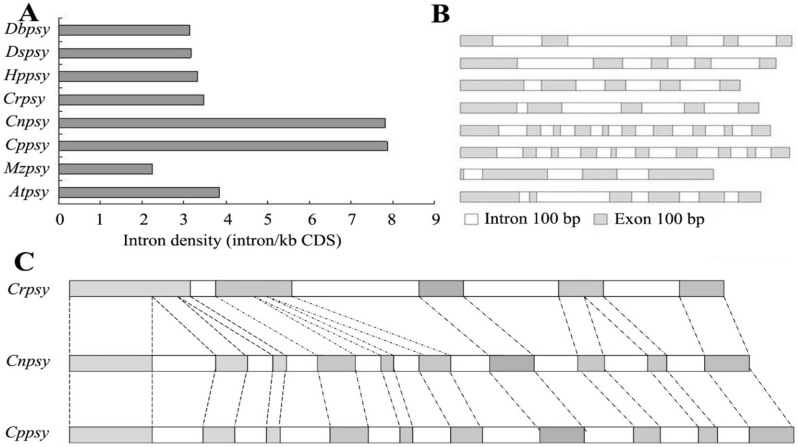
Exons and introns of the *Cppsy* gene in *C. protothecoides* CS-41.The ten exons are: (1) 1 bp to 280 bp; (2) 477 bp to 576 bp; (3) 691 bp to 743 bp; (4) 913 bp to 1030 bp; (5) 1139 bp to 1180 bp; (6) 1327 bp to 1427 bp; (7) 1635 bp to 1793 bp; (8) 1957 bp to 2045 bp; (9) 2171 bp to 2233 bp; (10) 2351 bp to 2488 bp. (**A**) Intron density; (**B**) DNA structure; (**C**) The relationship between introns and exons of *Cppsy*, *Crpsy*, and *Cnpsy* genes. *Dbpsy*, *Dspsy*, *Hppsy*, *Crpsy*, *Cnpsy*, *Cppsy*, *Mzpsy*, and *Atpsy* are the *psy* genes of *Duanliella bardawil*, *Duanliella salina*, *Haematococcus pluvialis*, *Chlamydomonas reinhardtii*, *Chlorella* NC_64A, *Chlorella protothecoides* CS-41, *Zea mays*, and *Arabidopsis thaliana*, respectively.

Analysis of the *Cppsy* gene structure (Figure 1) revealed that it is more complicated than those of dicot and monocot plants. It consists of ten exons and nine introns. *Chlorella* has a higher intron density than other algae and higher plants; in most of the higher plants, *psy* genes always have four or five introns, but this alga has nine introns. Compared with the structure of the *psy* gene from *C. reinhardtii* (*Crpsy*), it seems that there are two introns inserted into each of the first and second exons, and one intron inserted into the fourth exon, which makes the gene structure more complicated (Figure 1C).

### 2.2. Sequence Alignment and Phylogenetic Reconstruction

**Figure 2:**
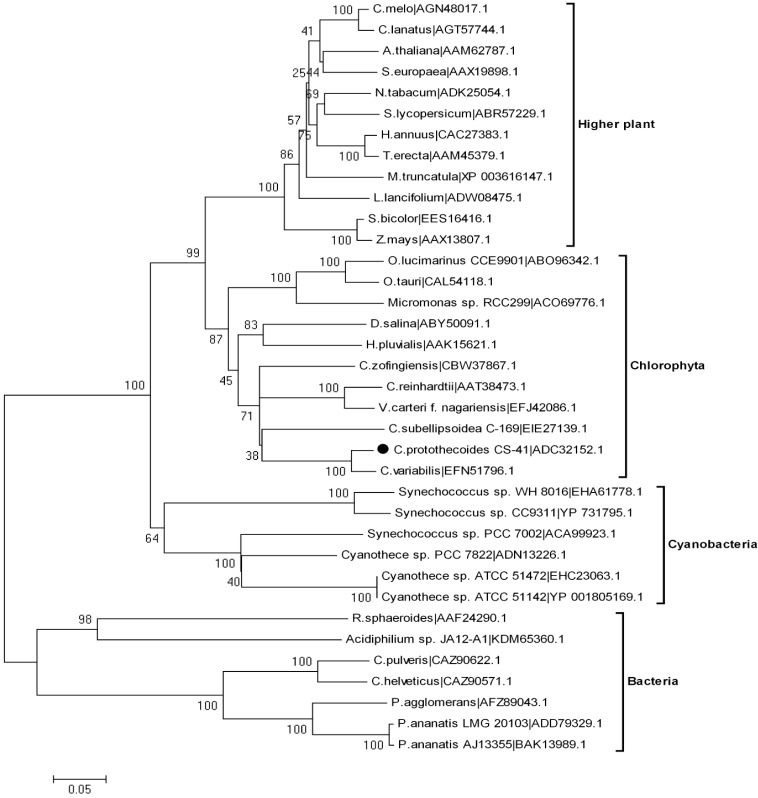
Phylogenetic tree of PSY sequences from various species. The phylogeny was derived using neighbor-joining analysis. The accession numbers of the amino acid sequences follow the taxon names. Horizontal branch lengths represent relative evolutionary distances, with the scale bar corresponding to 0.05 amino acid substitutions per site.

After the DNA and cDNA sequences of the *Cppsy* gene were determined, it was possible to investigate its evolutionary position among the various *psy* genes. Using MEGA 4.0 from Clustal W1.6 alignments, the phylogenetic tree of PSYs from different organisms was constructed based on their deduced amino acid sequences. It showed that *psy* was derived from an ancestor gene and later evolved into four subgroups, including higher plants, cyanobacteria, algae, and bacteria (Figure 2). According to the neighbor-joining (NJ) tree, *Cppsy* belongs to the algae group, and is more ancient than plant species (Figure 2).

**Figure 3:**
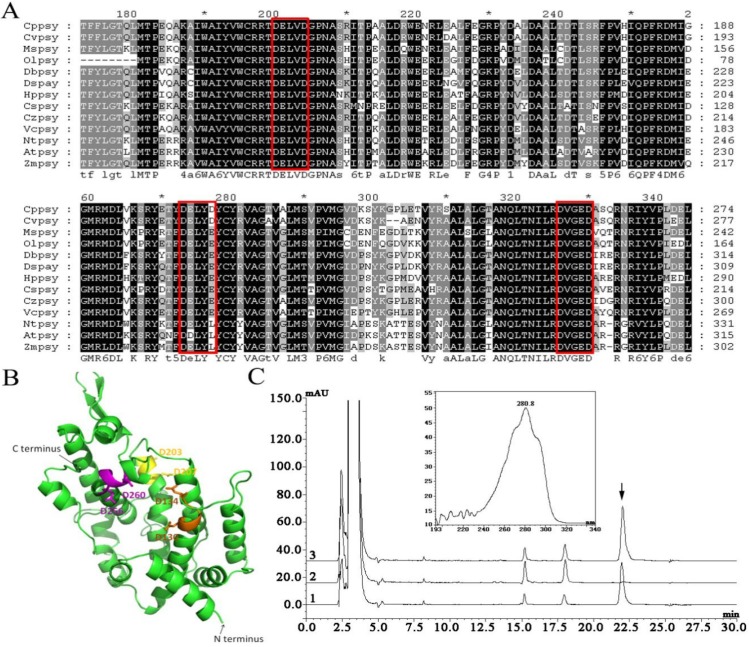
(**A**) Alignment of the selective PSY-deduced amino acid sequences from different algae produced with the GeneDoc program using Clustal W. The alignment indicates aspartate-rich regions/substrate-Mg^2+^ binding sites (DXXXD). The three DXXXD motifs are shown by the red boxes. Cppsy, Cvpsy, Mspsy, Olpsy, Dbpsy, Dspsy, Hppsy, Cspsy, Czpsy, Vcpsy, Ntpsy, Atpsy, and Zmpsy are the PSY of *Chlorella protothecoides* CS-41, *Chlorella variabilis*, *Micromonas* sp. RCC299, *Ostreococcus lucimarinus*, *Duanliella bardawil*, *Duanliella salina*, *Haematococcus pluvialis*, *Coccomyxa subellipsodiea* C-169, *Chromochloris zofingiensis*, *Volvox carteri* f. *nagariensis*, *Nicotiana tabacum*, *Arabidopsis thaliana*, and *Zea mays*, respectively; (**B**) Three-dimensional model structure of CpPSY. Comparative modeling was performed using homology-based three-dimensional structural modeling. The three aspartate-rich motifs (DXXXD) are colored in orange (DELVD), yellow (DELYD), and magenta (DEGED); others are shown in green. The *N*-terminus and *C*-terminus are also shown; (**C**) High-performance liquid chromatography trace and UV spectrum of carotenoid pigments in the *E. coli* heterologous complementation system. Pigments extracted from *E. coli* cells transformed with pACCRT-E and pUC-psy together (1), pUC-psy only (2), and pACCRT-EB only (3). Absorbance was recorded at 285 nm. The peak indicated by the arrow is phytoene.

The deduced amino acid sequence of *Cppsy* was submitted to NCBI for PSI-BLAST searches and the results showed that *Cppsy* has high homology with *psy* genes from other algal species, with 83% identity and 88% positives with *psy* from *Chlorella* NC_64A. *Cppsy* was also highly similar to *psy* from *C. reinhardtii* (67% identities, 79% positives), *H. pluvialis* (63% identities, 77% positives), *D. bardawil* (68% identities, 80% positives), and *D. salina* (68% identities, 79% positives), suggesting that *Cppsy* belongs to the algae *psy* family. In the algae family, CpPSY belongs to class I of PSY according to Tran’s data [29]. BlastP analysis suggested that this protein has the essential characteristics of PSY. It belongs to the Isoprenoid_Biosyn_C1 superfamily, and contains the consensus sequence, including three predicted substrate-Mg^2+^ binding sites (aspartate-rich regions) (DXXXD), 130-DELVD-134, 203-DELYD-207, and 256-DEGED-260 (Figure 3A). In other algae and higher plants, there are two (DELVD and DVGED) (Figure 3A); hence, CpPSY has one more DXXXD motif than other PSYs. The abundant 203-DELYD-207 site possibly plays an important role in the function of CpPSY, which should be studied further.

The secondary structure prediction carried out at NPS@ (https://npsa-prabi.ibcp.fr/) showed that CpPSY consists of 58.68% alpha helix, 26.58% random coil, 10.79% extended strand, and 3.95% beta turn. The tertiary structure of CpPSY was constructed using homology-based modeling by Swiss-Model (Figure 3B). A total of 50 models were found. Squalene synthase (HpnC) was used as a template for molecular modeling, since the identity is the highest (30.42%). The modeled structure also showed that CpPSY consists mostly of alpha helices. The three conserved DXXXD motifs (orange DELVD, yellow DELYD, and magenta DEGED) were marked in the three-dimensional model structure (Figure 3B). It seems that the three DXXXD motifs form a circle-like structure, which could be important for enzyme activity.

All of the analysis results strongly suggest that PSY from *C. protothecoides* CS-41 is an algal phytoene synthase protein involved in the carotenoid biosynthesis pathway. Bacterial complementation assay further confirmed that this gene is functional. The expressed protein in pUC-psy could catalyze the GGPP produced by pACCRT-E (Figure 3C,1) to phytoene, similar to the function of the *crtB* gene in pACCRT-EB (Figure 3C,3).

### 2.3. Promoter Isolation and Analysis

**Figure 4:**
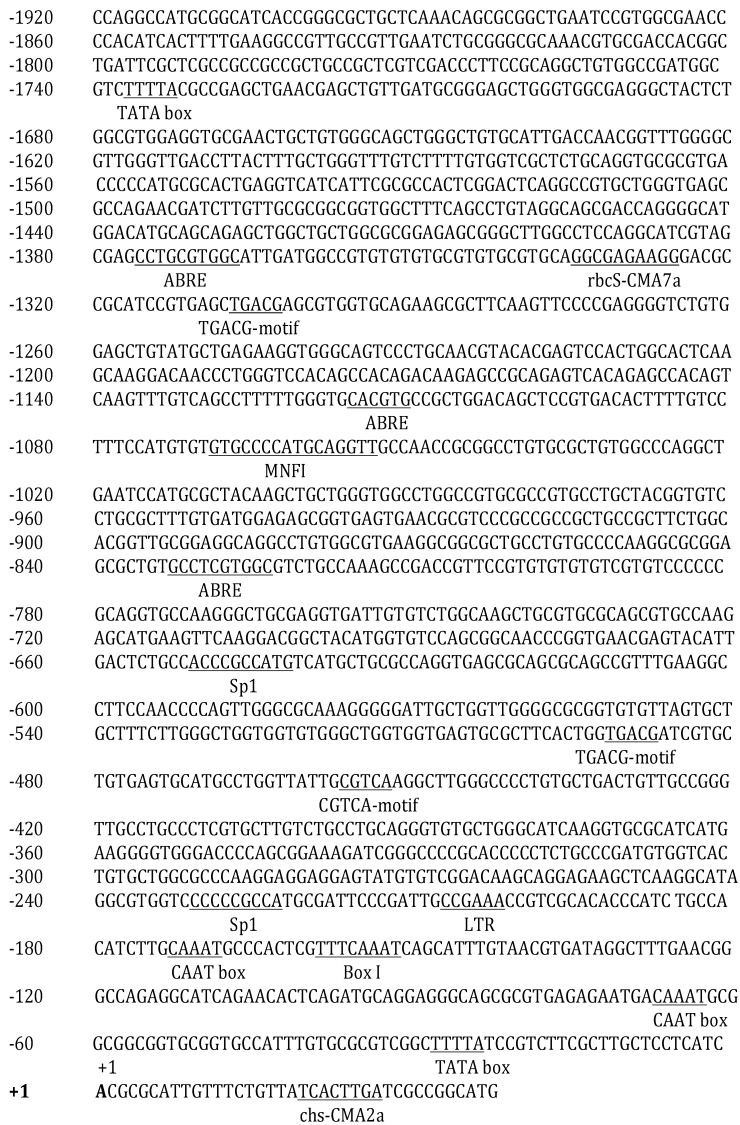
Promoter sequence of *Cppsy* from *C. protothecoides* CS-41. Numbers indicate the positions relative to transcriptional start site (TSS). The TSS is indicated as +1 and in bold; important *cis*-elements are underlined.

The promoter region of the *Cppsy* gene was cloned from *C. protothecoides* CS-41 genomic DNA. The cloned *Cppsy* promoter region was determined to be 1980 bp in length, and the sequence is shown in Figure 4. Furthermore, the cloned *Cppsy* promoter region was analyzed using the PLACE and PlantCARE databases. Several core fragments were identified, which are homologous to the *cis*-acting elements of higher plants and of great importance for the promoter functions (Figure 4). Three types of elements, which have been found to be regulated by hormones in the upstream region of some plant genes, are present in the *Cppsy* promoter: the ABRE type (CCTGCGTGGC, CACGTG, and GCCTCGTGGC) involved in abscisic acid responsiveness; the TGACG-motif (TGACG), the *cis*-acting regulatory element involved in methyl jasmonate (MeJA) responsiveness; and the Sp1 (CCCCCGCCA and ACCCGCCATG), MNF (GTGCCCCATGCAGGTT) and Box I (TTTCAAA) types involved in light responsiveness.

The transcriptional start site (TSS) of the *Cppsy* promoter was determined by 5′-RACE using total RNA extracted from *C. protothecoides*. The TSS is an adenine (A) at 34 bp upstream of the translation initiation codon. The distance between the putative TATA-box and TSS is approximately −24 to −28 bp, which is consistent with the distance of 32 bp ± 7 bp from previous data [30].

A previous study showed that the *psy* expression level is affected by light [31] and other biotic and abiotic stresses [16] in higher plants. Gene expression response to environmental stress is related to the regulation mechanism. To understand more about the regulation mechanism, we need to know more information about the gene, including the gene structure and regulatory domains. Here, many elements were found in the *Cppsy* promoter that belong to light-responsive elements, such as Sp1, MNF1, G-box, and chs-CMA2a. There were also some *cis*-acting elements involved in abscisic acid (ABRE) and MeJA (TGACG-motif) responsiveness. Light is one of the most important environmental factors for algae. To determine whether these motif sequences from the *Cppsy* promoter are involved in light, abscisic acid, and MeJA signaling, loss-of-function analysis needs to be carried out in future studies.

### 2.4. Gene Expression Response to Light and MeJA

**Figure 5:**
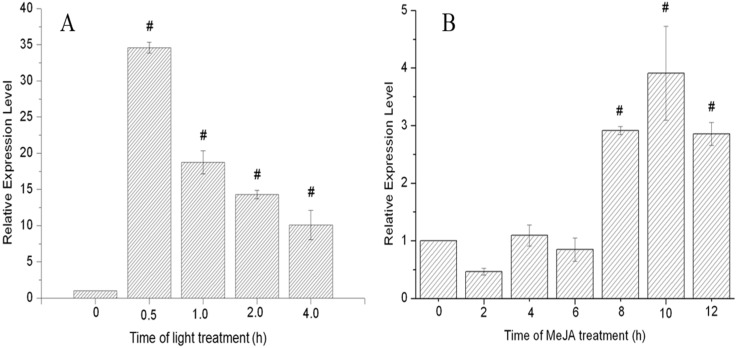
The expression of *Cppsy* gene induced by light (**A**) and MeJA (**B**) at different time points in *C. protothecoides* CS-41. Data (mean ± SEM) are combined from three independent experiments. # indicates that the gene expression levels were significantly different from that at 0 h (*p* < 0.01).

To investigate the effects of light and MeJA on the promoter inducibility at different time points, we analyzed *Cppsy* mRNA expression levels after light and MeJA treatment. The results show that on treatment with light, *Cppsy* gene expression increased by up to 36 times, compared with the dark, which indicates that *Cppsy* gene expression is up-regulated in response to light (Figure 5A). When treated with MeJA, *Cppsy* gene expression peaked at 10 h after treatment (Figure 5B). Therefore, the gene expression of *Cppsy* is significantly induced by treatment with light and MeJA (*p* < 0.01). These results confirm that the *Cppsy* promoter is induced by light and MeJA, and can be used as a candidate promoter element for the genetic modification of carotenoid biosynthesis in *Chlorella*, other algae, or higher plants.

## 3. Experimental Section

### 3.1. Strains and Culture Conditions

The microalgal strain used in this study was *C. protothecoides* CS-41 obtained from CSIRO Marine Laboratories (Hobart, Australia). They were grown in modified basal medium [32] containing 10 g·L^−1^ glucose at 28 °C and 180 rpm, and were collected at the log phase or late log phase.

### 3.2. Genomic DNA and RNA Isolation

Genomic DNA was extracted using a modified cetyltrimethylammonium bromide (CTAB) method [33]. The total RNA was isolated from *C. protothecoides* CS-41 cells at the late log phase (about 5-day incubation, with a cell density around 1 × 10^8^ cells mL^−1^) using TRIzol^®^ reagent (Invitrogen, Carlsbad, CA, USA) according to the manufacturer’s instructions.

### 3.3. Cloning of Full-Length Cppsy cDNA and Its Corresponding Gene

Degenerate primers (Table 1) were designed for the amplification of a partial *Cppsy* cDNA from *C. protothecoides* CS-41. The primers were derived from the highly conserved nucleotide and amino acid sequences reported for the *psy* genes from five kinds of algae (*C. reinhardtii*, *H. pluvialis*, *D. salina*, *D. bardawil*, and *Chlorella* NC_64A).

Sets of specific primers were synthesized based on the sequence of putative insert for 5′- and 3′-RACE [34]. YFO1 and YRI1 were used for 5′-RACE, and YFO2 and YRI2 (Table 1) were used for 3′-RACE. RACE was performed using the 5′-Full RACE Kit and 3′- Full RACE Core Set Version 2.0 (TaKaRa, Dalian, China) according to the manufacturer’s protocol. The RACE products were gel purified and sequenced as previously described. One pair of specific primers, YF1 and YR1 (Table 1), was designed from the sequences of the 5′- and 3′-RACE fragments to amplify the full-length *Cppsy* cDNA and its corresponding gene.

### 3.4. Southern Blot Analysis

According to the *Cppsy* genomic DNA sequence, *Bam*HI, *Eco*RI, *Nco*I, *Sma*I, and *Xma*I, which showed no recognition sites in the probed region of the *Cppsy* gene, were chosen to digest the whole genomic DNA. The probe was prepared by amplifying genomic DNA with the primers psy-F and psy-R, resulting in a 552-bp fragment of the *Cppsy* gene. The digested DNA was transferred to a Hybond-N membrane (GE Healthcare, Little Chanfont, UK) by capillary transfer and hybridized with a ^32^P-labelled DNA probe at both low and high stringency overnight.

After hybridization, the radioactivity of the membrane was monitored using a Storm 840 Phosphor Imaging System (Molecular Dynamics, Sunnyvale, CA, USA).

### 3.5. Bioinformatics Analysis

Comparative and bioinformatic analyses of the nucleotide sequences and deduced amino acid sequences were carried out online at http://www.ncbi.nlm.nih.gov and http://cn.expasy.org. The nucleotide sequence, deduced amino acid sequence, and open reading frame (ORF) were analyzed, and sequence comparison was conducted through database searches using BLAST programs (http://www.ncbi.nlm.nih.gov/BLAST/) and GeneDoc software. The phylogenetic analysis of *psy* from other plant species was aligned with Clustal X program version 1.83 using default parameters [35] and manual adjustments where necessary. A phylogenetic tree was constructed using MEGA (molecular evolutionary genetics analysis) program, version 4.0 [36] from Clustal W1.6 alignments. The NJ [37] method was used to construct the tree. In the NJ method, the P distance was used to analyze the amino acid sequences. A total of 1000 repetitions were performed using the bootstrap method to determine the reliability of each node of the tree. The homology-based three-dimensional structural modeling of PSY was accomplished using Swiss-Model and WebLab Viewer Lite (http://swissmodel.expasy.org).

### 3.6. Functional Complementation Experiment in E. coli

*E. coli* JM109 (Table 2) was used as a host for complementation experiments by cotransformation of the plasmid pUC-psy with pACCRT-E (Table 2). *E. coli* JM109 harboring only plasmid pACCRT-EB (Table 2) was cultured as a positive control, and only plasmid pUC-*psy* was cultured as a negative control for PSY functional analysis. The different strains were cultivated in 100 mL LB medium containing 100 µg·mL^−1^ ampicillin and 50 µg·mL^−1^ chloramphenicol at 28 °C and 180 rpm. IPTG (1 mM) was added when the optical density at 600 nm (OD_600_) reached 0.5, and the culture was kept at 28 °C for 2 days. The *E. coli* cells were collected by centrifugation at 12,000 rpm and used for high-performance liquid chromatography (HPLC) analysis.

**Table 2:** Strains and plasmids used in this study.

Strains or Plasmids	Characteristics	Source
*E. coli* JM109	Host for expression vectors	MOST-USDA Joint Research Center for Food Safety stock
pACCRT-E	pACYC184 containing *crtE* gene of *Erwinia uredovora* (Cm^r^) (metabolite: GGPP)	Gift from Prof. Gerhard. Sandmann (J.W. Goethe University, Frankfurt, Germany)
pACCRT-EB	pACYC184 containing *crtE* and *ctrB* genes of *Erwinia uredovora* (Cm^r^) (metabolite: Phytoene)	Gift from Prof. Gerhard. Sandmann (J.W. Goethe University, Frankfurt, Germany)
pUC19	Expression vector (Amp^r^)	MOST-USDA Joint Research Center for Food Safety stock
pUC-psy	pUC19 vector containing *Cppsy* gene (Amp^r^)	This work

Pigments in the bacteria were extracted according to procedures described by Breitenbach *et al.* [38]. *E. coli* JM109 cells harboring different plasmids were collected by centrifugation and freeze dried. Pigment extraction was carried out in acetone (80%, v/v) using ultrasonication, and the solvent was removed by blowing with nitrogen gas. The carotenoids were then resuspended in acetone for subsequent HPLC analysis. All operations were carried out on ice under dim light to prevent photodegradation, isomerizations, and structural changes of the carotenoids. The samples were prepared for HPLC by dissolving the dried residues in 1 mL of acetone and filtered through a polycarbonate 0.22 μm filter (Millipore, Carrigtwohill, Ireland). The extracted pigments were separated on a Kromasil KR100-5C_18_ analytical column (250 mm × 4.6 mm, 5 μm) using an UltiMate3000 HPLC system (Thermo Fisher Scientific, Waltham, MA, USA). The procedures described by Huang [39] were used with the following modifications: the mobile phase consisted of solvent A (acetonitrile/methanol/0.1 M Tris-HCl (pH 8.0), 84:2:14, v/v/v) and solvent B (methanol/ethyl acetate, 68:32, v/v). Pigments were eluted at a flow rate of 1 mL·min^−1^ with a linear gradient from 100% solvent A to 100% solvent B over a 5 min period, followed by 25 min of solvent B. The column temperature was maintained at 30 °C and the sample volume was 20 μL. The pigments were monitored by diode array detector, and the targeted products were identified by their absorption spectra and typical retention times compared with the control.

### 3.7. Promoter Isolation and Analysis

The Genomic Walking Kit (TaKaRa, Dalian, China) was used to obtain promoter regions of the *Cppsy* gene from *C. protothecoides* CS-41. Based on the *Cppsy* genomic sequences, gene-specific primers were designed and are listed in Table 1. Primary and nested PCRs were performed with the *Cppsy* gene-specific primers and Genome Walking adapter primers (AP1) in the kit according to the manufacturer’s instruction. The primary nested PCR products were diluted to 1:50 with distilled water for subsequent nested PCR. The nested PCR products were purified from 1.2% (w/v) agarose gel and sub-cloned into the pMD18T vector (TaKaRa, Dalian, China). The cloned vectors were then sequenced and the putative *cis*-regulatory elements were analyzed using the PLACE [40] and PlantCARE databases [41].

### 3.8. Gene Expression Response to Light and MeJA

To analyze the light regulation pattern of the *Cppsy* gene in *C. protothecoides*, algal cells in the late log phase were cultivated in the dark for more than 2 days, then collected by centrifugation at 5000 rpm for 15 min in the darkness. The pellet was resuspended in fresh medium without glucose, and then subjected to light treatment under a light intensity of 120 μmol m^−2^·s^−1^ for different induction times (0, 0.5, 1.0, 2.0, and 4.0 h). Each treatment was carried out with three parallel repetitions.

To analyze the MeJA regulation pattern of the *Cppsy* gene in *C. protothecoides*, algal cells in the log phase were treated with 100 μM MeJA (Sigma, St. Louis, MO, USA) diluted in dimethyl sulfoxide (DMSO) for 0, 2, 4, 6, 8 10, and 12 h. Control cultures were treated with DMSO only.

The effects of light and MeJA on the *Cppsy* gene transcripts in *C. protothecoides* were quantified by reverse transcriptase quantitative PCR (RT-qPCR). The RT-qPCR experiment was performed in two steps: the cDNA templates were synthesized from RNA samples using Prime Script™ Reverse Transcriptase Reagent according to the manufacturer’s instructions (TaKaRa, Dalian, China) using oligo (dT) as the primer; then qPCR was conducted on an iQ Cycler (Bio-Rad, Watford, UK) using the specific primers (Table 1) and the SYBR ExScript RT-PCR kit (TaKaRa, Dalian, China). The specific primers for the corresponding genes included psyRT-F and psyRT-R for the *Cppsy* gene, and 16SRT-F and 16SRT-R for the 16S gene (Table 1). Before the qPCR expression analysis, we checked the amplification efficiency of each primer pairs, and all were well controlled between 99.83% and 101.25%.

Each qPCR measurement was carried out independently at least three times, and the mean value was used for quantification. The 2^−ΔΔCT^ method was used to analyze the relative changes in gene expression, the expression of the 16S gene was used as a normalized control, and the expression of the untreated samples was used as a negative control.

## 4. Conclusions

Carotenoid pathways in plants have been described in great detail using genetic, biochemical, and molecular data, mainly from *Arabidopsis* and other higher plants; however, this is the first study in the unicellular microalga *C. protothecoides* CS-41.

We successfully isolated and analyzed the *Cppsy* gene, which encodes the functional phytoene synthase—a vital enzyme for carotenoid biosynthesis in *C. protothecoides* CS-41—as well as its promoter. Computational analysis suggested that this protein belongs to the Isoprenoid_Biosyn_C1 superfamily. It contains one more substrate-Mg^2+^ binding site than other algae and higher plants. Analysis also demonstrated several candidate motifs for the promoter, which exhibited light- and MeJA-responsive characteristics. Light- and MeJA treatment showed that the *Cppsy* expression level was significantly enhanced by light and MeJA.

These achievements will be helpful to understand more about the regulatory mechanism of the carotenoid biosynthesis pathway in algae and the mechanisms for accumulation of lutein and other important carotenoids.

## Supplementary Files

Supplementary File 1
