# Supplementary material for: Isolation and Analysis of the Cppsy Gene and Promoter from Chlorella protothecoides CS-41
Source: Mar Drugs. 2015 Oct 28;13(11):6620–35. doi: 10.3390/md13116620 (PMC4663545; doi:10.3390/md13116620)
Supplement: Supplementary File 1 [file marinedrugs-13-06620-s001.docx]

**Supplementary Information**


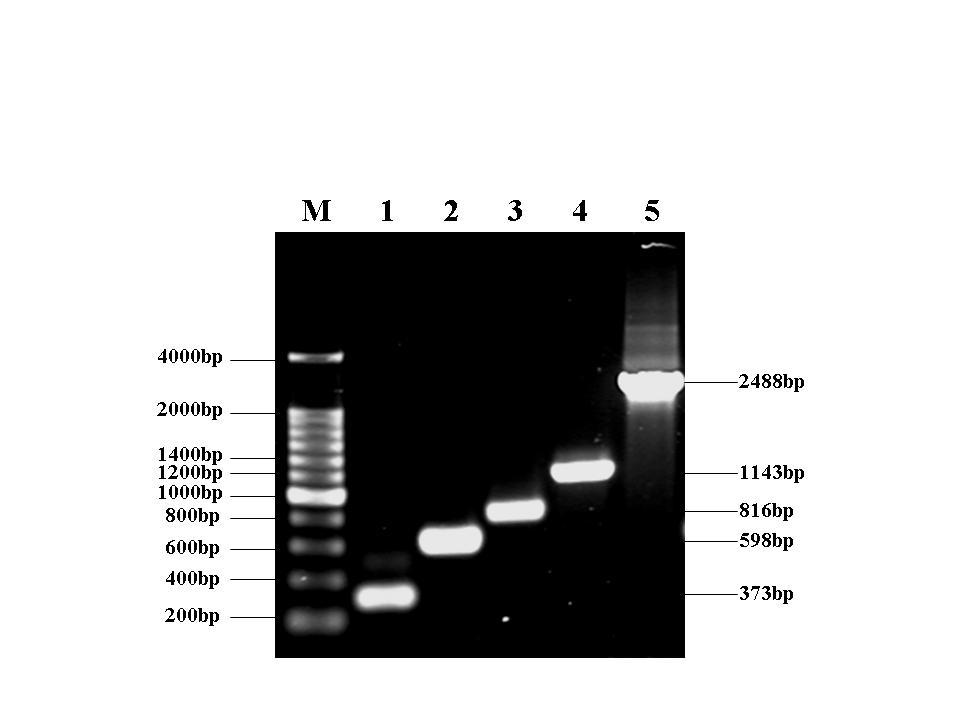


**Figure S1.** The electrophoresis of high fidelity PCR products of the *Cppsy* gene.
M: 200 bp DNA Ladder; (**1**) Core fragment; (**2**) 5′RACE product; (**3**) 3′RACE product;
(**4**) *Cppsy* cDNA fragment; (**5**) *Cppsy* DNA fragment.


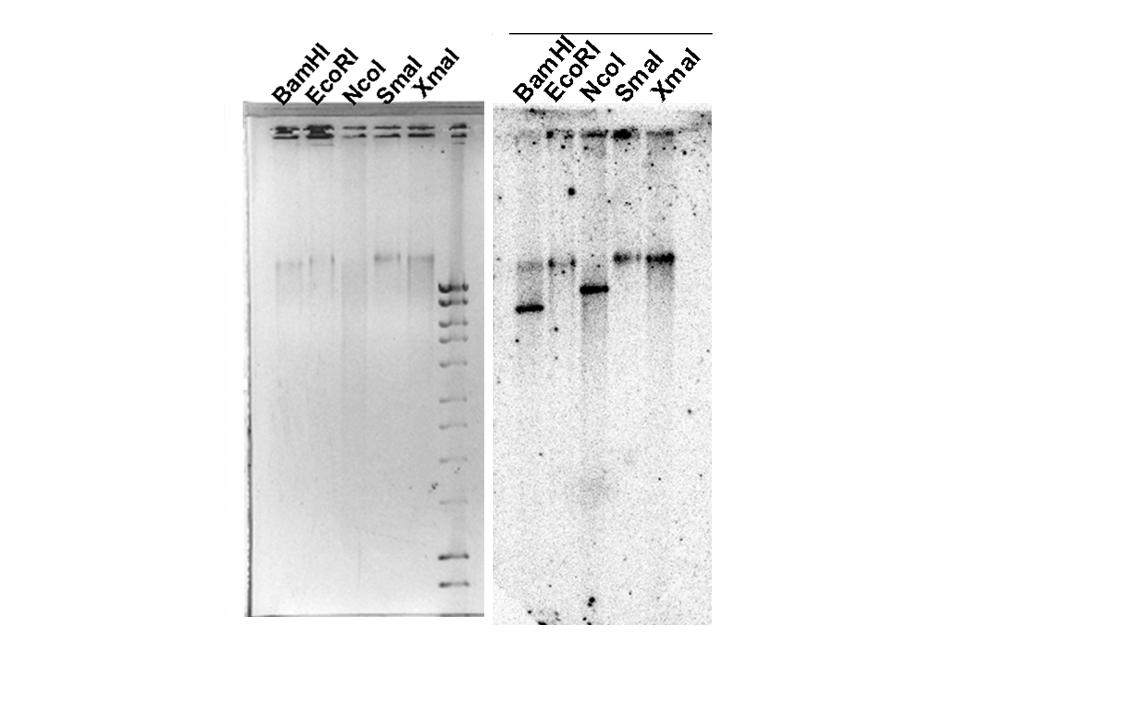


**Figure S2.** Southern blot analysis of the *Cppsy* gene in *C. protothecoides* CS-41.
